# Broadening of Neutralization Activity to Directly Block a Dominant Antibody-Driven SARS-Coronavirus Evolution Pathway

**DOI:** 10.1371/journal.ppat.1000197

**Published:** 2008-11-07

**Authors:** Jianhua Sui, Daniel R. Aird, Azaibi Tamin, Akikazu Murakami, Meiying Yan, Anuradha Yammanuru, Huaiqi Jing, Biao Kan, Xin Liu, Quan Zhu, Qing-an Yuan, Gregory P. Adams, William J. Bellini, Jianguo Xu, Larry J. Anderson, Wayne A. Marasco

**Affiliations:** 1 Department of Cancer Immunology & AIDS, Dana-Farber Cancer Institute; Department of Medicine, Harvard Medical School, Boston, Massachusetts, United States of America; 2 National Center for Infectious Diseases, Centers for Disease Control and Prevention, Atlanta, Georgia, United States of America; 3 State Key Laboratory for Infectious Disease Prevention and Control and National Institute for Communicable Disease Control and Prevention; Chinese Center for Disease Control and Prevention, Changping, Beijing, China; 4 Department of Medical Oncology, Fox Chase Cancer Center, Philadelphia, Pennsylvania, United States of America; University of California Irvine, United States of America

## Abstract

Phylogenetic analyses have provided strong evidence that amino acid changes in spike (S) protein of animal and human SARS coronaviruses (SARS-CoVs) during and between two zoonotic transfers (2002/03 and 2003/04) are the result of positive selection. While several studies support that some amino acid changes between animal and human viruses are the result of inter-species adaptation, the role of neutralizing antibodies (nAbs) in driving SARS-CoV evolution, particularly during intra-species transmission, is unknown. A detailed examination of SARS-CoV infected animal and human convalescent sera could provide evidence of nAb pressure which, if found, may lead to strategies to effectively block virus evolution pathways by broadening the activity of nAbs. Here we show, by focusing on a dominant neutralization epitope, that contemporaneous- and cross-strain nAb responses against SARS-CoV spike protein exist during natural infection. *In vitro* immune pressure on this epitope using 2002/03 strain-specific nAb 80R recapitulated a dominant escape mutation that was present in all 2003/04 animal and human viruses. Strategies to block this nAb escape/naturally occurring evolution pathway by generating broad nAbs (BnAbs) with activity against 80R escape mutants and both 2002/03 and 2003/04 strains were explored. Structure-based amino acid changes in an activation-induced cytidine deaminase (AID) “hot spot” in a light chain CDR (complementarity determining region) alone, introduced through shuffling of naturally occurring non-immune human VL chain repertoire or by targeted mutagenesis, were successful in generating these BnAbs. These results demonstrate that nAb-mediated immune pressure is likely a driving force for positive selection during intra-species transmission of SARS-CoV. Somatic hypermutation (SHM) of a single VL CDR can markedly broaden the activity of a strain-specific nAb. The strategies investigated in this study, in particular the use of structural information in combination of chain-shuffling as well as hot-spot CDR mutagenesis, can be exploited to broaden neutralization activity, to improve anti-viral nAb therapies, and directly manipulate virus evolution.

## Author Summary

The SARS-CoV caused a worldwide epidemic of SARS in 2002/03 and was responsible for this zoonotic infectious disease. The role of neutralizing antibody (nAb) mediated immune pressure in the evolution of SARS-CoV during the 2002/03 outbreak and a second 2003/04 zoonotic transmission is unknown. Here we demonstrate nAb responses elicited during natural infection clearly have strain-specific components which could have been the driving force for virus evolution in spike protein during intra-species transmission. *In vitro* immune pressure using 2002/03 strain-specific nAb 80R recapitulate a dominant escape mutation that was present in all 2003/04 animal and human viruses. We investigated how to generate a single broad nAb (BnAb) with activity against various natural viral variants of the 2002/03 and 2003/04 outbreaks as well as nAb escape mutants. Remarkably, amino acid changes in an activation-induced cytidine deaminase (AID) “hot spot” of somatic hypermutation and localized to a single VL CDR were successful in generating BnAbs. These results provide an effective strategy for generating BnAbs that should be generally useful for improving immune based anti-viral therapies as well as providing a foundation to directly manipulate virus evolution by blocking escape pathways.

## Introduction

A novel coronavirus (CoV), severe acute respiratory syndrome coronavirus (SARS-CoV), caused a worldwide epidemic of SARS with a fatality rate of 9.6% in 2002/03 and later reemerged and resulted in infection of four individuals with full recovery in the winter of 2003/04 [1]–[5]. SARS-CoV has been demonstrated to be a zoonotic disease that evolved in palm civet and human hosts. The global outbreak that occurred in 2002/03 and the cluster of 2003/04 SARS cases were the result of two independent zoonotic transfers from palm civets to humans [6]–[9]. Although palm civets were identified as the hosts involved in human transmission, evidence suggested the existence of another precursor reservoir. Indeed bats, predominantly horseshoe bats, were later found to be a natural reservoir of SARS-like-CoVs, and harbor more diverse viruses than any other hosts [10]–[14]. Variants of SARS-like-CoVs circulating in bats may cross the species barrier again and this threat is enhanced by the large numbers of bats that often congregate, their broad geographic distribution and their ability to travel long distances. Diversity of host range and variant immune pressures within the natural reservoir or intermediate hosts are likely to continue to drive SARS-CoV evolution.

Phylogenetic analyses have provided clear evidence that amino acid changes in spike (S) protein of animal and human viruses obtained during and between the two zoonotic transfers were the result of positive selection. These studies suggested that the S gene underwent strong positive selection for the adaptation to human hosts during the interspecies transmission; a positive selection pressure during transmission within same species was also clearly demonstrated [6]–[8]. The role that nAb-mediated immune pressure played in driving the positive selection, particularly during intra-species transmission, is still unknown.

Over the last several years, neutralizing human monoclonal antibodies (mAbs) have been developed as potential therapeutics for the prophylaxis and treatment of SARS [15]–[20]. NAbs-mediated protection can also prevent the escape of mutant viruses from cytotoxic T-lymphocytes that are commonly associated with rapid disease progression and severity [21],[22]. Although there has not been a recent SARS-CoV outbreak, it is desirable to develop effective Ab-based passive immunotherapy for this zoonotic respiratory pathogen that might continue to evolve under immune pressure within the animal kingdom and has the potential to rapidly adapt in humans.

We previously developed a potent human nAb 80R against S protein of both civet and human 2002/03 viral strains that demonstrated profound protection against viral infection in a SARS-CoV mouse model. Our studies revealed that 80R recognizes a conformationally sensitive epitope located within the receptor binding domain (RBD) [23]. A comprehensive neutralization sensitivity/resistance profile for 80R was also established based on the detailed epitope mapping and a co-crystallographic structural study [23],[24]. In this paper, we used this nAb and detailed knowledge of its epitope to examine nAb responses in convalescent serum samples from chronically exposed civet farmers, 2002/03 and 2003/04 SARS outbreak patients, and 2004 civet cats. Our studies provide the first evidence that contemporaneous strain-specific and cross-strain nAb responses against S protein are present in natural civet cat and human infection. *In vitro* neutralization escape studies with 80R recapitulated a highly conserved escape mutation that also occurred naturally from 2002/03 to 2003/04 viruses regardless of host species. The structural features of human nAbs that were required to broaden their activity to include binding to escape mutants was also investigated. Indeed, among several different approaches examined, amino acid changes in a single activation-induced cytidine deaminase (AID) “hot spot” of a light chain CDR alone, introduced through shuffling of naturally occurring non-immune human variable light chains (VLs) or by targeted CDR mutagenesis were sufficient to generate broad nAbs (BnAbs) against a range of viral strains including neutralization escape variants. These results have important implications for the management of new emerging viral pathogens and provide a strategy to directly manipulate virus evolution through Ab blockade of escape pathways.

## Results

### Serologic analysis

**Figure 1:**
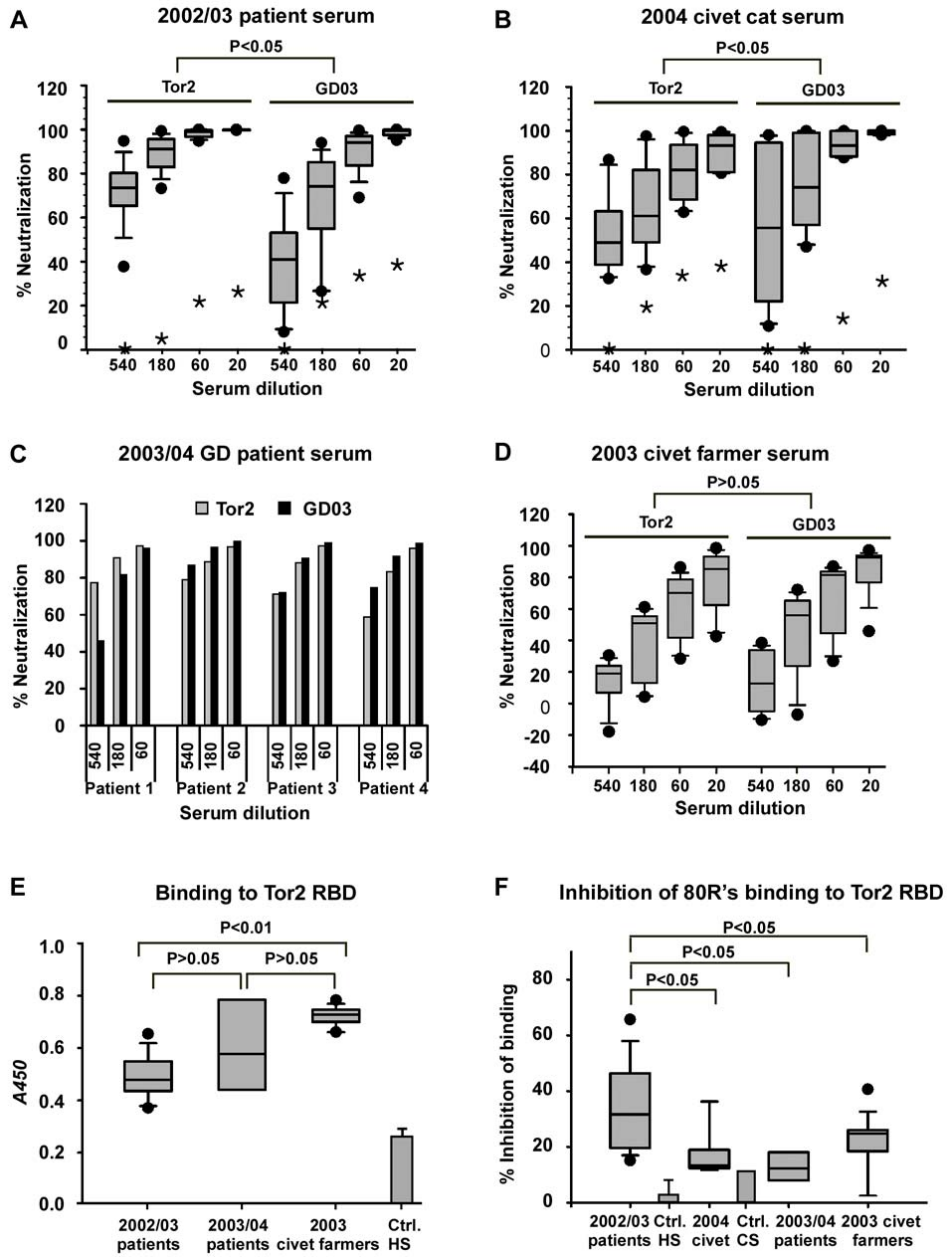
Serologic analysis. A, 11 of convalescent serum samples were obtained from SARS patients of the 2002/03 outbreak in China. Neutralizing activities of these serum samples were analyzed against pseudotyped viruses bearing the S protein of Tor2 or GD03 at indicated dilutions. Data were shown in a box and whiskers graph. The box extends from 25th percentile to the 75th percentile, with a line at the median. The whiskers above and below the box indicate the 95th and 5th percentiles. The dots above and below the box showed highest and lowest data points. A symbol of “*” indicates the data points of a SARS-CoV negative healthy human serum sample. IC90 for each patient serum was calculated and the statistic analysis was done with IC90 value using one way ANOVA for correlated samples. The same data presentation and the statistic analysis methods were used for the following panels B and D. B, Neutralization assay of Tor2 and GD03 pseudotyped viruses with six civet serum samples collected from animal market in Guangzhou in Jan. 2004. Similarly as Fig. 1A, “*” indicates the data points for a SARS-CoV negative civet cat serum sample. C, Neutralization assay of Tor2 and GD03 pseudotyped viruses with four serum samples from 2003/04 outbreak. The samples labeled in the graph as patient 1–4 that were the same patients described before [5]. D. Ten serum samples from civet cat farmers collected in June 2003in Guangdong Province were analyzed against Tor2 and GD03 pseudotyped viruses. E, Tor2 -RBD binding activity of 2002/03, 2003/04 outbreak, and Civet cat farmers' serum samples were analyzed by ELISA. These serum samples were the same as those used in Panel A, C, and D, respectively. Dilution of serum was 1∶180. The data was presented the same way as panel A and unpaired Student t-test was used for statistical analysis. The average and standard deviation (SD) of background binding of two SARS-CoV negative human serum samples (Ctrl.HS) were also shown. F, A comparison of the competition ability of different serum samples for 80R's binding to Tor2-RBD were evaluated by ELISA. Dilution for all samples was 1∶20. The non-specific competition for 80R's binding to Tor-RBD by two Ctrl. HS or one control civet cat serum (Ctrl.CS) was also shown. Serum samples were corresponding to those used in panel A, B, C or D. The data presentation and statistic analysis were done the same way as panel E.

Eleven randomly selected serum samples from patients who developed SARS during the 2002/03 outbreak were analyzed for their neutralizing activities against S protein pseudotyped viruses. The S protein from Tor2 and GD03 viral strains that were used are representative of the late phase of the 2002/03 outbreak and 2003/04 human cases, respectively. Phylogenetic analysis of different viral isolates from human patients and civets of the two epidemics demonstrated a close relationship of Tor2 and SZ3 (2003 Civets); while GD03 is closer to PC04 (2004 Civets) [8]. Though all 11 serum samples from 2002/03 outbreak were able to neutralize Tor2 and GD03 pseudotyped viruses, the potency was quite different (Fig. 1A). The 2002/03 patient serum samples are statistically significantly more potent in neutralizing Tor2 than GD03. In contrast, statistical analysis of 2004 civet cat sera showed higher neutralizing activity against GD03 compared to Tor2 (Fig 1B). In addition, neutralization activity was found in three of the four 2003/04 outbreak patient sera to be slightly higher against GD03 strain compared to Tor2 (Fig. 1C). Interestingly, surveillance sera samples collected from civet cat farmers in June 2003, a period between the 2002/03 and 2003/04 outbreaks, showed the same neutralizing titers to Tor2 and GD03, likely owing to their chronic exposure to the SARS-like-CoVs (Fig. 1D). The Tor2-RBD binding activity of these serum samples was also tested. Unexpectedly, there was higher binding activity of Tor2-RBD with 2003 civet cat farmers' serum than was seen with 2002/03 patient sera (Fig. 1E). A further comparison of the ability of different serum samples to compete for 80R binding to Tor2-RBD showed that the 2002/03 patient sera competed for 80R's binding significantly stronger than did each of the 2003/04 serum samples (Fig. 1F). Taken together, these two latter observations suggest that a larger percentage of Tor2-RBD directed Abs in 2002/03 serum samples are “80R-like” nAbs than in 2003/04 serum samples. These results also demonstrate that contemporaneous-strain and cross-strain nAb are produced in both humans and animals following natural SARS-CoV/SARS-like-CoV infection.

### Neutralization escape from 80R

Molecular evolution studies on SARS-CoVs during the 2002/03 outbreak and between the two zoonotic transfers have provided evidence of positive selection pressure in the S gene [8],[9]. *In vitro* neutralization escape studies using the 2002/03 strain specific nAb 80R were performed to simulate humoral immune pressure *in vivo*. After incubation of plaque purified SARS-CoV (Urbani used here, same as Tor2) with 80R, a total of 4 isolated plaques were picked, viable viruses were obtained from all of these four plaques after three passages in cell culture. All viruses were confirmed to be 80R-resistant at a concentration 100-fold greater than that needed for neutralizing 90% of wild-type viruses. Sequencing of the complete S gene of these four 80R-escape variants revealed one common mutation at amino acid position 480. Three of the S variants carried a mutation of lysine to alanine (D480A), and the other one had a mutation of lysine to glycine (D480G). Although four other mutations were also shown in the S protein, none of them appeared more than once in the four escape variants and 3 of them were located outside the RBD (Table 1). We have previously described that D480A/G change completely abolished binding of 80R to S protein or RBD fragment (S318-510) [23] and D480 is the key residue which forms a negative charged binding interface patch with 80R [24]; mutation of 480 did not affect binding of RBD of S protein to human ACE2 receptor [25] or viral replication in Vero cells (data not shown). Therefore it is clear that the D480A/G mutation sufficiently confers 80R resistance without cost or gain on viral fitness in human hosts. Importantly, the D480G mutation arose from the selection pressure of 80R that coincides with the change from D480 in S proteins of 2002/03 viruses (SZ3/Tor2) to G480 in 2003/04 viruses (PC04/GD03) (Table 2).

**Table 1:** Amino acid sequences in spike protein of 80R neutralization escape mutants.

		Amino acid position				
		82	228	292	421	480
**Wild type (Urbani)**		P	I	S	L	D (gat)
**80R escape mutants**	**Iso1-100**	P	I	G	L	G (ggt)
	**Iso1-101**	P	I	S	L	A (gct)
	**Iso1-102**	S	T	S	F	A (gct)
	**Iso1-103**	P	I	S	L	A (gct)

**Table 2:** Amino acid differences in the RBD of spikes.

	Representative strain name	GenBank Accession No.	Amino acid position					
			344	360	472	479	480	487
**2002/2003 human outbreak**	Tor2	AY274119	K	F	L	N	D	T
**2002/2003 Civet**	SZ3	AY304486	R	S	L	K	D	S
**2003/2004 human outbreak**	GD03	AY525636	R	S	P	N	G	S
**2003/2004 Civet**	PC04	AY613951	R	S	P	R	G	S

### Selection for BnAbs by *de novo* human non-immune scFv library panning against variant S proteins

Having obtained evidence of naturally occurring human and animal 80R-like nAbs and of a dominant 80R neutralization escape pathway that appeared to occur during natural SARS/SARS-like CoV infection, we sought to isolate new BnAbs with pan-activity against 2002/03 and 2003/04 strains as well as 80R escape variants and to identify structural features that were unique and/or common for their broad activity. Signature amino acid differences in S protein at positions 472 and 480 between 2002/03 and 2003/04 strains provided a finite way to interrogate the breadth of nAb binding and neutralization activity (Table 2).

#### Identification of nAb 11A

**Figure 2:**
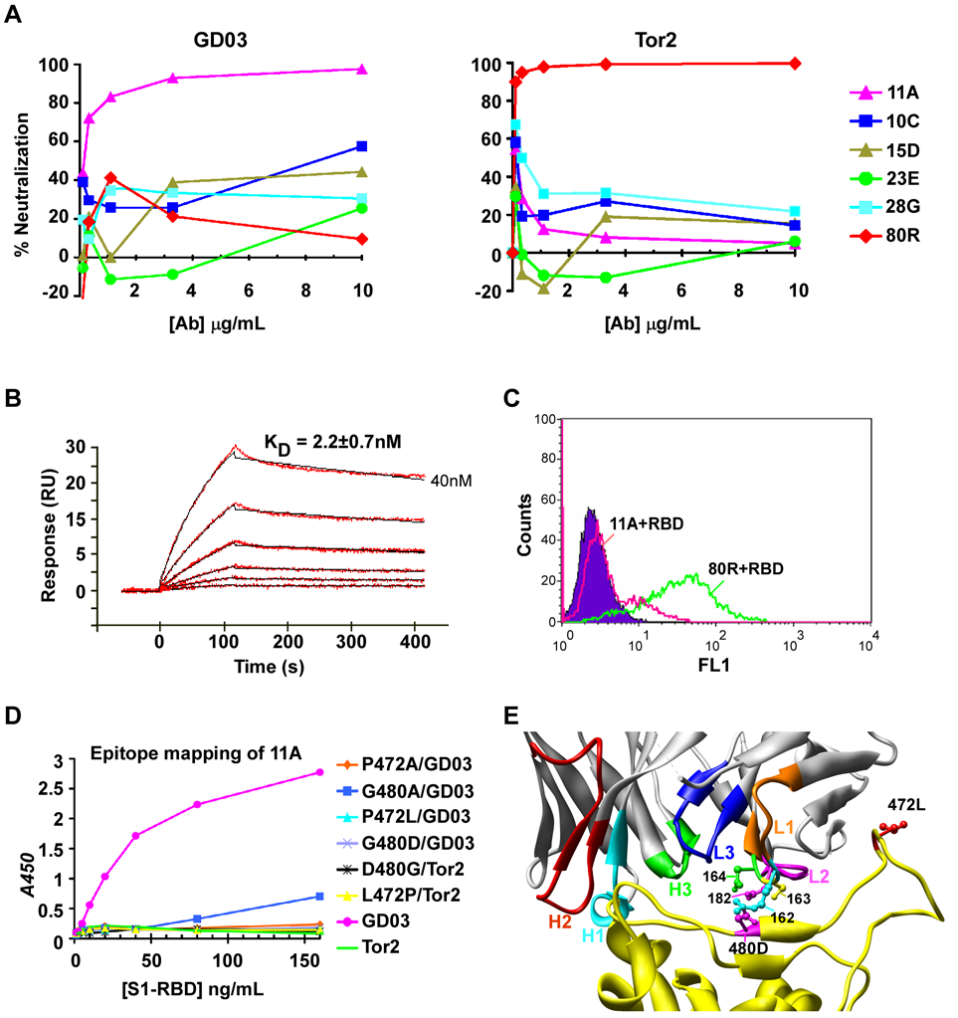
Neutralization of pseudotyped viral infection by anti-GD03 Abs and epitope mapping of GD03 nAb 11A. A, Five anti-GD03 Abs isolated by phage Ab library screening against purified GD03-RBD were analyzed for neutralizing activity using GD03 (Left panel) or Tor2 (Right panel) spike pseudoyped viruses. B, Kinetic characterization of GD03-RBD binding to 11A-IgG1. Abs were captured on a CM4 chip via immobilized anti-human IgG1 on the chip. GD03-RBD at various concentrations (2 folds serial dilutions, highest concentration was indicated) was injected over the chip surface. Binding kinetics was evaluated using a 1∶1 interaction model. In each panel, the binding response curves (red lines) are overlaid with the fit of the interaction model (black lines). All *ka*, *kd*, *KD* value showed in the table represent the means and standard errors of three experiments. C, Competition of 11A for the binding of GD03-RBD-C9 to 293T-ACE2 cells. GD03-RBD-C9 or control-RBD-C9 (Filled purple) used for staining is at 20 ug/mL and the Abs (256 or 80R negative control) were used at 50 ug/mL to compete for the binding of GD03-RBD to 293T-ACE2 cells. D, Epitope mapping of 11A. Purified proteins of a set of GD03-RBD or Tor2-RBD mutants were coated to ELISA plates at indicated concentrations. 2 µg/ml of 11A-IgG1 followed by HRP-anti-human IgG1 were used to detect the binding of 11A with different mutants. E, Interface of structure of the Tor2- RBD/80R complex ^21^. S1-RBD is in yellow. CDR loops (H1-H3 and L1-L3) of 80R, amino acids of 80R-CDRL1 and L2 (162–164 and 182) as well as 472L and 480D of S1-RBD are colored as shown.

GD03-RBD was initially used as bait to isolate cross-strain nAbs that could recognize promiscuous amino acids at positions 472 and 480. Five unique antibodies (11A, 10C, 15D, 23E, 28G) were identified by panning against GD03-RBD-C9 with two non-immune human Ab phage display libraries and neutralizing activity was tested against pseudotyped viruses. One of them 11A was found to be a potent nAb against GD03 but not against Tor2 (Fig. 2A). Kinetic analysis of 11A IgG binding to GD03-RBD demonstrated high affinity interaction (K_D_ = 2.2±0.7 nM) (Fig. 2B). 11A also potently inhibited GD03-RBD's binding to ACE2 expressing 293T cells (Fig. 2C). Epitope mapping showed that two amino acids in the GD03-RBD region (472P and 480G) were critically important for 11A's binding. Single amino acid changes at either position resulted in complete loss of 11A binding (Fig. 2D). Other amino acid changes at positions of V404A, Y442F, K465A, T468A and S487A did not affect 11A binding (data not shown). On the basis of this result, all of the 2004 civet viruses (PC04) should also be recognized and neutralized by 11A since PC04 has same 472P and 480G as GD03 (Table 2). In contrast to 11A, 80R was only affected by changes at position 480 but not 472 [23]. As shown in Fig. 2E, 472 is located on a ridge formed by an extended loop that is reinforced by the Cys^467^-Cys^474^ disulfide bond [26]. This suggests that 11A recognizes a slightly different neutralizing epitope since the 80R CDR residues do not make contact with this segment of RBD [24].

#### Identification of an anti-D480A Ab, 256

**Figure 3:**
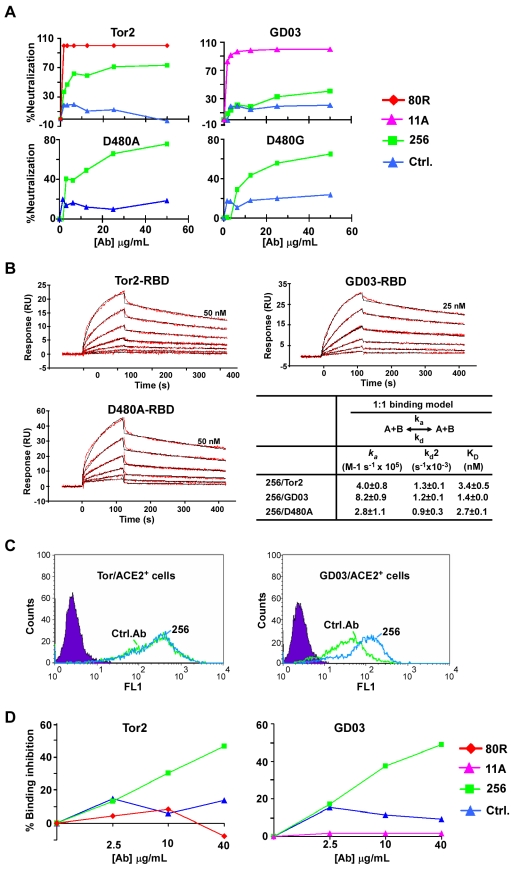
Characterization of Ab 256. A, Neutralization of pseudoviral infection by 256-IgG1. An anti-CXCR4 Ab 33-IgG1[42] was used as a negative control, 80R and 11A were used as positive control for Tor2- and GD03-viruses, respectively. B, Kinetic characterization of the binding of spike RBDs to 256-IgG1. Binding kinetics was evaluated similarly as described in Fig. 2. B. C, Competition of 256 for the binding of Tor2- or GD03-RBD-C9 to 293T-ACE2 cells. Left, competition for the binding of Tor2-RBD-Ig to 293T-ACE2 cells. 0.5 ug/mL of Tor2-RBD-Ig or control-Ig (Filled purple) used for the staining of 293T-ACE2 cells and the scFvs (control or 256) were used at 5 ug/mL to compete for the binding. Right, competition for the binding of GD03-RBD to 293T-ACE2 cells. The assay was the same as Fig. 2C except 256 was used here. D, Ab 256 Competition ELISA Assay. A fixed amount of 256 scFv expressing phages (256-phages) were mixed with various scFv-Fc antibody or full-length IgG1s at indicated antibody concentration, and the mixtures were then added to Tor2-RBD (left) or GD03-RBD (right) coated ELISA plate. The competition of 256-IgG1s for the binding of 256-phages to RBDs were determined by measuring the remaining binding of 256-phages using HRP-anti-M13 antibody. 256-phages homologous to 256-IgG1 were used as positive controls and both showed competition for 256-phage binding to Tor2-RBD and GD03-RBD, 80R or 11A did not show inhibition of 256-phages binding to either Tor2- or GD03-RBDs.

In a parallel effort D480A- or D480G-Tor2 RBD coated on immmunotubes and conjugated to magnetic beads were used as panning targets together with a third non-immune scFv library to isolate the desired BnAbs. Only panning against D480A-magnetic beads resulted in D480A-RBD specific ELISA positive clones and only one clone, scFv 256 bound to cell-surface expressed D480A full-length S protein and showed neutralization activity against D480A S pseudotyped viruses (data not shown). 256-scFv was converted to whole human IgG1 and then tested separately for neutralization of Tor2-, GD03-, D480A-, and D480G-pseudotyped viruses. As shown in Fig. 3A, 256-IgG1 neutralized D480A and D480G (D480A>D480G) but with <80% activity at the highest Ab concentration tested (50 µg/mL). It also inhibited Tor2 viral entry but was much less potent than 80R and only marginally neutralized GD03. Furthermore, 256-IgG1 showed even lower neutralization titer in a micro-neutralization assay (Table 3) using Urbani (Tor2 equivalent) wild type and 80R escape viruses which may be explained, at least in part, by the conformational differences between the recombinant S protein and the S on the pseudotype or natural virus. Interestingly, kinetic studies showed that 256-IgG1 had high binding affinity with all three RBDs: Tor2-, GD03- and D480A-RBD (Fig. 3B). While this discrepancy between high binding affinity of Ab 256 and weak pseudo/micro neutralization activity could also be due to conformation difference of S, alternatively it could be based on 256's mechanism of neutralization which is not by direct competition of S binding to ACE2. As shown in Fig. 3C, Ab 256 does not compete with Tor2-RBD for cell-surface ACE2 receptor binding (left panel), but dramatically augmented GD03-RBD's binding with ACE2 (right panel). As expected the 256 epitope is also distinct from 80R and 11A, neither 80R nor 11A competed with 256 for Tor2- (Fig. 3D, left) or GD03-RBD binding (Fig. 3-D, right) in a competition ELISA assay. Thus, BnAbs against 80R escape mutant D480A/G as well as 2002/03 and 2003/04 strains were not isolated through numerous *de novo* variant S protein pannings using non-immune Ab-phage libraries.

**Table 3:** Microneutralization (MNt) assay.

Abs*	MNt titers**	Virus
80R	2560	Wild type SARS-CoV (Urbani)
11A	<20	
**256**	**20**	
80R	<20	80R escape mutant Iso1-100 (D480G)
11A	<20	
**256**	**40**	
80R	<20	80R escape mutant Iso1-103(D480A)
11A	<20	
**256**	**40**	

*: Whole IgG1 was used for all antibodies in this assay.

**: Reciprocals of the highest dilutions of Abs that showed less than 50% cytopathic effects in 2 of the 3 wells tested.

### A structure-based approach to broadening 80R's neutralization spectrum through light chain shuffling and focused mutagenesis

**Figure 4:**
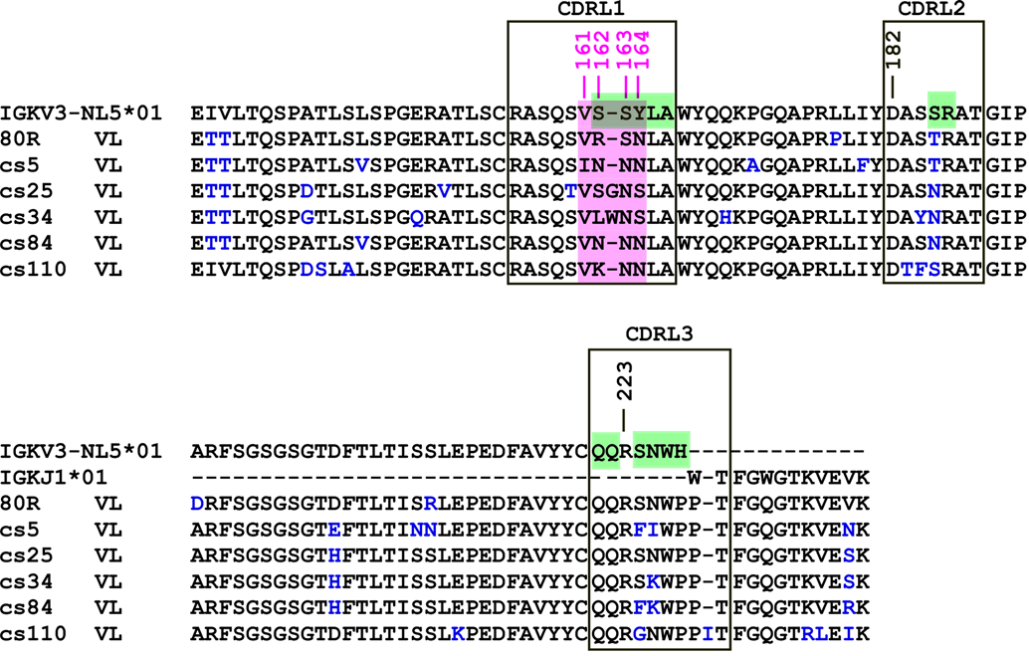
Sequence alignment of the Vk of five Abs identified from 80R-Vk-cs library and 80R. The best-matched germline Vκ and Jκ genes of 80R are showed on top of the alignment. CDR regions are labeled in large boxes, amino acid substitutions from germline are colored in blue. A dash indicates no amino acid at that position. Amino acids 161–164 in CDRL1 were highlighted in pink. All 5 Abs have one consensus change from S to N at position 163. WRCY hot spots of AID are colored in green.

Structural data guided a different approach to engineer 80R to have more broadly neutralizing activity against D480A/G and 2003/04 outbreak strains. The co-crystal structure of the Tor2-RBD in complex with 80R shows that D480 lies at the center of RBD-80R interface. In addition, all of the D480 contacting residues are located in the Vκ light chain. In particular in 80R CDRL1, D480 makes an intermolecular salt bridge to R162 that is flanked by two neutral residues: V161 and S163, and an H-bond to N164 [24]. Other contacting residues are D182 in CDRL2 and R223 in CDRL3. Notably, amino acids 162–164 form part of a WRCY “hot spot” motif for AID-mediated somatic hypermutation (SHM) [27],[28] and R162 and N164 are mutated from germline serine (Fig. 4). This suggested firstly that natural mutations within this hot spot would likely exist in our circa 10^8^ member non-immune V*kappa* (Vκ) repertoire and secondly, that focused mutagenesis on this “hot spot” would also provide an experimental system to test whether mutations within this region would broaden binding and neutralization activity. Accordingly two directed approaches, Vκ light chain shuffling (cs) and CDRL1 (amino acids 161–164) focused mutagenesis (fm) were simultaneously utilized to identify natural or directed variation in critical Vκ contact amino acids, respectively.

Both 80R-Vκ-cs and two 80R-fm phage display libraries were constructed and selected against different RBDs (Table 4). Only clones that bound to four variant S protein RBDs (Tor2, Tor2-D480A/G, GD03) were further characterized. Five unique Abs were identified in the 80R-Vκ-cs studies following panning against D480A-RBD. Remarkably, a common feature of 5 unique Abs recovered is the amino acid changes in CDRL1 region from position aa161–164 that are important contact residues for D480 in RBD. One consensus change in all cs mutants is S163N at position 163 (Fig. 4). These 5 Abs maintained germline D182 in CDRL1 of IGKV3-NL5*01 and germline R223 in CDRL3 of IGKJ1*01, respectively. These results provide further evidence for the critical importance of these CDRL1 contact residues in spike protein binding specificity. In addition, the finding that these Abs also selectively used VLs originating from the same rearranged IGKV3-NL5*01 germline gene as the parental Ab 80R (Fig. 4) suggests that this type of VL structure may provide a critical “pattern recognition” motif for this epitope which is necessary to create the functional binding site of 80R and its derivatives.

**Table 4:** 80R-cs and 80R-fm libraries and their selection results.

Library		Library size	Panning antigen conjugated on beads	Positive clone screening (“+”/total clones tested) *	Unique “+” clones for further characterization
Vκ-cs		6.3×10^6^	D480A-RBD	154/196	cs-5, cs-25, cs-34, cs-84, cs-110
fm	161–163	1.5×10^4^	D480A-RBD	77/96	fm4,fm5, fm6,fm12
			D480G-RBD	48/96	fm39
	162–164	3×10^4^	GD03-RBD	47/96	fm4,fm5, fm6,fm12,fm39

*: Two rounds of selection for 80R- Vκ-cs library and one round selection for 80R-fm library were performed before ELISA screening for positive clones.

**Figure 5:**
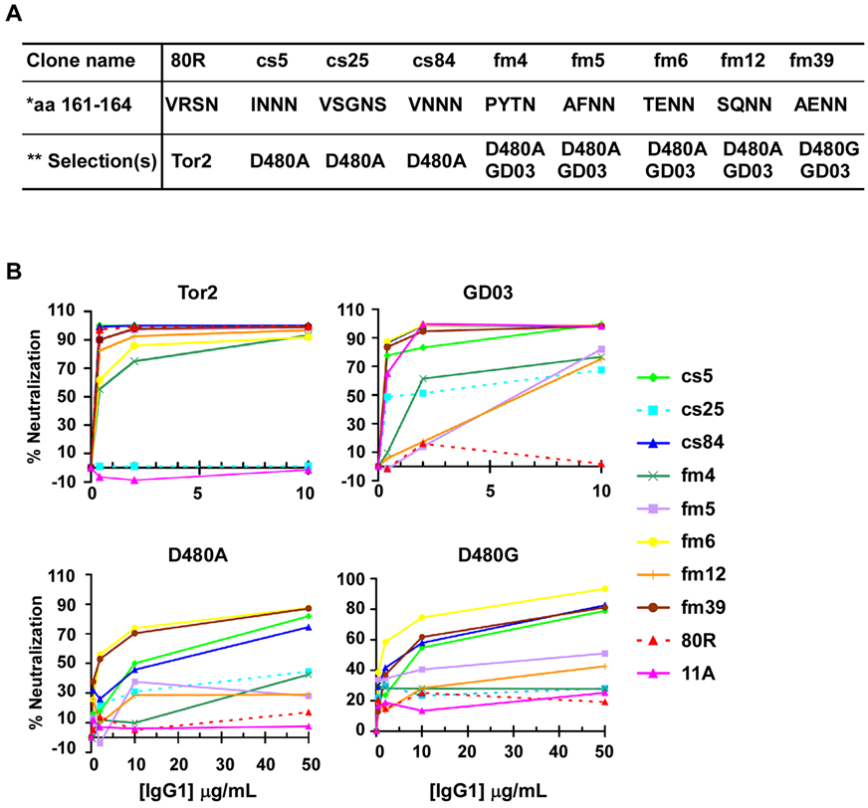
Broadly neutralizing activity of cs- and fm-Abs. A, sequence comparison of eight Abs identified from 80R-cs or -fm library selection. *Amino acid sequence in CDRL1 (161–164). ** 80R was identified from Tor2-S1 targeted library selection [17]; five fm-Abs were from both D480A or D480G and GD03 targeted library selection. B, Neutralization activity of the eight Abs (full-length IgG1) was tested with the same pseudotyped viruses as used in Fig. 3A at indicated Ab concentration. Cs5, cs84, fm6 and fm39 appeared to be the four BnAbs.

The 80R-fm libraries were panned against three RBD targets, D480A, D480G and GD03 and five clones that were positive by ELISA for all four targets (including Tor2) were chosen for further characterization. The amino acid sequences in CDRL1 (161–164) of these 5 clones were shown in Fig. 5A. Four of these fm Abs were identified from D480A and GD03 and one (fm39) from D480G and GD03 panning. Consistent with the results from the 80R-Vκ-cs studies, four of these fm Abs had the S→N change at position 163 and all five maintained the 164N mutation found in parental 80R. Thus, this “hot spot” is indeed of central importance in controlling the breadth of binding activity.

Next, eight scFvs were converted to human IgG1 mAbs and tested for neutralization of pseudotyped viruses. As shown in Fig. 5A and B, among the three cs-Abs and five fm-Abs, R→N change at position 162 was associated with increased potency of cs5 and cs84 whereas R→E charge reversal at 162 was found with the most potent fm-Abs: fm6 and fm39. Other Abs, cs25, fm4, fm5 and fm12 showed less broad or weak neutralization activity. Thus, both cs- and fm- library strategies resulted in isolation of BnAbs with activity against four RBD variants.

### Kinetic changes on RBD binding resulting from broadening 80R's neutralization activity

Five most potent nAbs above (cs5, cs84, fm5, fm6, fm39) were evaluated further for their binding kinetics and affinity with various RBDs. The kinetic data obtained from binding of Tor2- or GD03-RBD to Ab-captured biosensor surfaces were evaluated using a 1∶1 binding model or a two state conformational change model (Table 5 and Fig. S1). For cs-Abs, the kinetic data fit 1∶1 binding model perfectly. The interactions of Tor2 with 80R or fm-Abs exhibited a double exponential pattern, which is not due to the heterogeneity of Tor2 and Abs, therefore the kinetic analyses of these Abs using two state conformational change model are presented. This suggests that a conformational change may occur after the formation of the initial binding complex. For the binding of GD03 to 11A and all the 80R's cs/fm mutants, kinetic parameters were derived from 1∶1 binding model. Due to the poorer response of the D480A to nAbs, kinetics could not be accurately derived; however, the affinity was determined by steady state affinity model and the biosensor-grams are presented for qualitative comparisons (Fig. S1).

**Table 5:** Kinetics of Ab interactions with Tor2-RBD or GD03-RBD and affinity of Abs binding with D480A-RBD.

1∶1 binding model 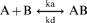				Two state conformational change model 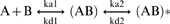					Affinity model
	*k*_a_ (M^−1^ s^−1^×10^5^)	*k*_d_ (s^−1^×10^−3^)	*K*_D_ (nM)	*k*_a_1 (M^−1^ s^−1^×10^5^)	*k*_d_1 (s^−1^×10^−2^)	*k*_a_2 (s^−1^×10^−3^)	*k*_d_2 (s^−1^×10^−3^)	*K*_D_ (nM)	*K*_D_ (µM)
80R/Tor2				9.3±4.8	5.8±3.4	7.3±0.8	0.4±0.0	3.2±0.3	
fm5/Tor2				4.4±2.4	3.3±0.2	6.0±0.4	0.3±0.0	4.0±1.2	
cs84/Tor2	1.2±1.1	5.0+2.8	5.2±1.7						
cs5/Tor2	7.7±5.7	4.3±2.0	6.4±2.1						
fm6/Tor2				2.9±0.3	6.2±0.0	13.0±0.0	1.2±0.0	17.6±0.0	
fm39/Tor2				2.5±1.5	9.8±3.4	14.3±4.1	1.8±0.0	51.2±21.0	
11A/GD03	3.6±1.3	0.7±0.7	2.2±0.7						
cs5/GD03	4.7±1.1	1.3±0.2	2.8±0.3						
cs84/GD03	3.3±0.4	1.0±0.0	2.9±0.4						
fm6/GD03	5.8±0.5	1.7±0.1	3.0±0.4						
fm39/GD03	7.7±0.3	2.8±0.5	3.6±0.5						
fm5/GD03	11.4±2.0	42.4±0.1	38.3±0.6						
fm39/D480A									0.2±0.0
fm6/D480A									0.2±0.0
cs5/D480A									0.5±0.1
fm5/D480A									1.1±0.3
cs84/D480A									1.4±0.2

The kinetic data were analyzed by using either the 1∶1 interaction model or the two state conformational change model. At least duplicate experiments were run for each binding interactions. The averaged value for each parameter, together with the standard error, is shown.

Table 5 summarizes the kinetics and affinities derived from different nAbs and models. Of note, the affinity of the cs5 and cs84 Abs for Tor2-RBD was≤one-fold lower than 80R however, both nAbs gained cross-reactivity for GD03, D480A and D480G. They also exhibit a similar high affinity as the potent GD03 nAb 11A for binding to GD03-RBD, and 0.2–1.4 µM affinity for binding to D480A-RBD to which neither 80R nor 11A can bind. By comparison, the fm6 and fm39 Abs have circa 10-fold lower affinity for Tor2-RBD, but they maintain high affinity binding for GD03 and are the highest affinity D480A binding Abs that were isolated.

## Discussion

Molecular phylogenic analyses have provided evidence that positive selection pressure was behind the evolution of SARS-CoV S gene during and between the two 2002/03 and 2003/04 epidemics [8],[9]. However, direct evidence that nAb-mediated immune pressure is one of the main driving forces of virus evolution, especially in intra-species transmission, is lacking. In this study we focused on a critical neutralization epitope and demonstrate that contemporaneous-strain and cross-strain nAb responses co-exist during natural SARS-CoV infection of civet cats and humans. In addition, *in vitro* nAb escape studies have provided strong support for the existence of a natural nAb driven evolution pathway. Moreover, structure-based 80R-VL shuffling and somatic hypermutation “hot spot” targeted mutagenesis were successful at generating BnAbs with activity against 80R escape mutants as well as 2002/03 and 2003/04 strains.

NAb responses were measured in convalescent serum from chronically exposed civet farmers, 2002/03 and 2003/04 SARS patients and 2003/04 civet cats against prototypic SARS-CoV strains Tor2 and GD03 that were representative of the two zoonotic transfers to humans. NAb levels in convalescent serum from 2002/03 SARS patients were higher against contemporaneous viral strain represented by Tor2 than against the 2003/04 GD03 strain. Similarly, 2004 civet cat serum had higher nAb levels against GD03 strain than against the 2002/03 Tor2 strain. In addition, a higher percentage of Tor2-RBD directed Abs in 2002/03 patient serum competed for the 80R epitope, as compared to chronically exposed, asymptomatic 2003 civet farmers serum which had similar nAb titers to both Tor2 and GD03 strains (Fig 1D–F). 2002/03 patient's serum samples also better competed for the 80R epitope than did each of the 2003/04 serum samples tested (Fig. 1E–F). These results demonstrated that cross-neutralization activity was present in all serum samples, suggesting that some neutralizing epitopes are conserved. Meanwhile, nAb responses elicited during natural infection clearly have strain-specific components. Indeed, 80R-like 2002/03 viral strain specific nAbs were found at higher levels in 2002/03 serum samples than 2003/04 samples from both animals and humans.

NAbs naturally derived from memory B-cells of a 2002/03 SARS patient that could neutralize both Tor2 and GD03 have been reported [15],[20],[29],[30]. Among the 23 such nAbs representing six functional groups based on virus strain cross-reactivity, the five nAbs in group III are most like 80R in their ability to only neutralize human isolates from the 2002/03 outbreak [30], although it is unknown if they have the same D480 dependence as 80R. NAbs similar to 11A were not identified probably because this donor was not exposed to the GD03 prototypic isolate with 472P/480G amino acids. Of particular interest is group VI that contained four BnAbs against all human and animal viruses from both outbreak strains. This study demonstrates that nAbs with different activities are produced in serum of naturally infected hosts and suggests that neutralization activity represents the sum of these polyclonal nAb responses. Importantly, while memory B cells producing BnAbs (e.g. Group VI) were elicited during natural SARS-CoV infection, they may contribute to only a small percentage (<20% - four out of total 23 nAbs) of the circulating nAbs [30]).

The potential role of human nAbs for protection against SARS-CoV infection has been established by several groups [15]–[19],[31]. As a consequence of high mutation rate of these RNA viruses, the activity of nAbs can be dramatically impacted by neutralizing escape variants of SARS-CoV. *In vitro* immune pressure with 80R, readily gave rise to escape variants at only one amino acid position (D480A or D480G) with the acquisition of an 80R-resistant phenotype and the mutation is neutral for viral replication or fitness in permissive cells. Position 480 has not been identified as a positively selected site for adaptation to or in human hosts [8],[32],[33]. However, the D480G mutation coincides with the change from D480 in S proteins of all 2002/03 viruses (Tor2 and SZ3) to G480 in all 2003/04 viruses (GD03 and PC04) (Table 2). NAb pressure by 80R-like Ab or Group III Abs which exists naturally could give rise to intermediate D480G escape mutants that may continue to evolve under immune pressure from SZ3/Tor2-like strains to PC04/GD03-like strains. Indeed, phylogeny analysis of S genes from 2003 civets (SZ3) and 2004 civets (PC04) indicates a positive selection during animal-to-animal transmission. Thus, in civets, 80R or 80R-like nAbs-mediated immune pressure could be a major driving force for positive selection of G480 in PC04 during evolution from SZ3 or a common ancestral strain (s).

Broadening the activity of human nAbs either naturally by B-cell hypermutation or synthetically through Ab engineering to include binding to escape mutants and additional viral strains could be one way to interfere with the viral evolution pathway and more efficiently control virus infection in humans. As a first step to isolating BnAbs with activity against 80R escape mutants as well as 2002/03 and 2003/04 strains, three large non-immune Ab-phage libraries were used to pan against variant RBD proteins. Since the GD03 shares the same 480G as 80R escape mutant, we hypothesized that selection with GD03-RBD may generate new nAbs, not only against GD03 but also 80R's resistance mutants. While nAb 11A was identified with high-affinity binding and potent neutralization to GD03, it did not neutralize other viral infections including Tor2, it's D480A/G mutants and SZ3 (data not shown). A similar rationale was employed to isolate BnAbs using D480A/G-Tor2-RBD as library selection. Only one Ab 256 that had extraordinary binding affinity for Tor2, D480A and GD03 but poor neutralization activity, particularly against the latter strain was isolated. Unlike 80R and 11A, nAb 256 did not compete for RBDs binding to ACE2. These results demonstrate that the strategy of *de novo* selection with non-immune libraries against different viral spike proteins is neither efficient nor sufficient in generating BnAbs with the desired extended spectrum of activity. Whether this limitation of *de novo* selection for BnAbs can be overcome by using immune libraries from SARS-CoV infected patients remains to be determined.

Structural data obtained from Tor2 RBD-80R scFv co-crystallographic studies provided a different approach to directly manipulate 80R to broaden its specificity as an alternative to antigen-driven passive selections. These studies indicated that only VL makes significant intermolecular contacts with D480. Notably, critical contact amino acids R162, S163 and N164 of CDR1 lie within a predicted “hot spot” for AID mediated SHM. While combinatorial light chain shuffling has been reported previously to provide diversity and a drift in viral epitope recognition [34],[35], a focus on a single VL CDR “hot spot” alone as an immune strategy to broaden nAb activity has not been reported. We therefore explored 80R-Vκ chain shuffling and focused mutagenesis of amino acids 161–164 as strategies to broaden the fine specificity of 80R and to overcome the resistance to D480A/G mutations. In both cases, only small libraries were found to be necessary to isolate novel nAbs with the desired properties (Table 4). Vκ shuffling library selection resulted in five new Abs against D480A, with three Abs, cs-5, cs-25 and cs-84 broadly neutralizing all four viral strains. Remarkably, sequence analysis revealed the common feature that all Abs had amino acid changes in the CDRL1 region from position aa161–164, with a consensus change of S→N at position 163. Potency of broad neutralization for cs-5 and cs-84 was further enhanced by the positive to neutral R→N change at aa162. Additionally, the fm-derived phage display libraries that carried saturated mutations for 80R CDRL1 amino acids 161–164 resulted in the isolation of five BnAbs with activity against the four viral strains. Importantly, the consensus S163N change was again found and parental 80R Y164N mutational change was maintained. Thus, the specificity of the high affinity and potent nAb 80R, that was originally targeted to SARS-CoV Tor2, was successfully broadened to become active against other viral strains including GD03 and 80R's escape mutants without compromising its original potency against Tor2. These studies suggest that even without having crystal structure information, selecting a chain (VH or VL) shuffled library against an escape mutant will likely provide important paratope information on regions which could account for the escape from parental Ab. Likewise, SHM hot-spot targeted mutagenesis strategy may be of similar great value when it is combined with a known structure.

Finally, the development of a single nAb or nAb combinations with sufficient breadth of protection against multiple viral strains including escape mutants and those that may arise by future zoonotic transfers is of great importance. One strategy, that we term “convergent combination immunotherapy” (CCI), focuses on applying intense nAb immune pressure on a single or overlapping neutralizing epitope such that neutralization escape is prevented or would occur at a great cost on viral fitness. Structural data on SARS-CoV evolution provide support for this concept in that certain mutations in S1 that overlap with the 80R epitope (e.g. N479K/R, T487S) result in a circa 20-fold loss in binding affinity for ACE2 [33]. Success at broadening nAb specificity to include a dominant D480A/G neutralization escape pathway is the first step in testing this important hypothesis where engineered broad nAbs such as 80R-fm6 and/or other 80R-cs/fm variants, could be used either alone or in combination to manipulate virus evolution and compromise fitness through Ab blockade of escape pathways. Other approaches like “divergent combination immunotherapy” (DCI), that target two or more non-overlapping neutralizing epitopes such as on S1 and S2, are not addressed in this work but represent another important tactic that could be used with the potent nAbs discovered here. In a similar manner to mAb therapies, vaccine strategies could be designed to produce BnAbs that recognize potential escape variants before they naturally occur. Indeed, inclusion of *in vitro* derived escape variants that can promote AID-mediated sequence diversification of germinal center B-cells is a vaccine strategy worth further investigation [36].

## Materials and Methods

### Serum samples

Convalescent serum samples from SARS patients during the 1^st^ SARS epidemics in 2003 were collected 90–120 days after the onset of symptoms from Inner Mongolia Autonomous Region, China. Four serum samples from 2003/04 sporadic cases were collected 11 days after onset of symptoms. Serum samples from healthy blood donors were used as negative controls. The diagnostic criteria for SARS-CoV infection followed the clinical description of SARS released by WHO. Ten serum samples from farmers were collected from a Civet cat farm in Zhaoqing, Guangdong Province in June 2003. Also included in this study were six civet serum samples collected from Xinyuan animal market in Guangzhou prior to culling in Jan. 2004, one sample collected from a SARS-like-CoV negative civet cat was used as a control [37]. All the serum samples were collected by China CDC virologists and were verified to be anti-SARS-CoV Ab positive as detected by enzyme-linked immunoabsorbent assay (ELISA) using commercially available diagnostic kits. Civet cat samples were verified positive for SARS-like-CoV by RT-PCR for the N and P genes. All of the sera were heat-inactivated at 56°C for 30 mins prior to performing the experiments.

### Neutralization assay with S-protein-pseudotyped lentiviruses

Full-length S gene of SARS-CoV Tor2 or GD03 was generated *de novo* by recursive PCR [23],[38]. S variants containing D480A or D480G mutant were generated by site-directed mutagenesis using S gene of Tor2 as template. Plasmids encoding full-length S protein of wild type or variant SARS-CoVs were constructed for making pseudotyped viruses. S-protein-pseudotyped lentiviruses expressing a luciferase reporter gene were produced as described previously [23],[39]. Briefly, 293T cells were co-transfected with a plasmid encoding full-length S protein variants, a plasmid pCMVΔR8.2 encoding HIV-1 Gag-Pol, and a plasmid pHIV-Luc encoding the firefly luciferase reporter gene under control of the HIV-1 long terminal repeat. Forty-eight hours posttransfection, viral supernatants were harvested for neutralization assay. Testing antibodies or sera at different dilutions were incubated with adequate amount of S-protein-pseudotyped viruses for 30 mins at room temperature (RT). The mixture was then added to ACE2-expressing 293T cells in 96 well plates. Infection efficiency was quantified by measuring the luciferase activity in the target cells with an EG&G Berthold Microplate Luminometer LB 96V.

### Generation and characterization of 80R neutralization escape variants

80R escape mutants were generated by incubating an equal volume (0.5 ml) of wild-type SARS-CoV (Urbani strain, 3×10^6^ pfu/ml) and 1.5 ug/ml of 80R Ab (giving 90% inhibition of viral infection) for 1 h at 37°C and 5% CO_2_. The virus-80R mixture were added into a monolayer of Vero E6 cells in 6-well plates and incubated with cells for 1 h at 37°C and 5% CO_2_, then the virus was removed and the cells were washed twice with DMEM medium. Finally, cells were overlaid with 2.5 ml of 5%FBS/DMEM culture medium containing the above concentration of 80R and 1% agarose, incubated for 3 days. 1 ml of 3% neutral red were added to each well, and left the plates overnight in 37°C/CO_2_ incubator. The next day, isolated escape virus plaques were picked and transferred into medium, freeze-thawed 3 times. The plaque-picked virus was propagated in Vero E6 cells in the presence of 80R for three passages until a cytopathic effect (CPE) was evident. The passaged viruses were then incubated with 80R in the plaque assay to confirm an 80R resistance phenotype and to generate the plaque-purified (subcloned) mutant viruses. The subclones of the escape virus mutant were then propagated, aliquoted and stored at −70°C. To identify possible mutations in the SARS-CoV spike protein of each of the escape viruses, viral RNA of each of the escape viruses and wild-type SARS-CoV virus was isolated and converted into cDNA by standard RT-PCR. The PCR products were cloned by TOPO-cloning vector (Invitrogen), and 5 clones of each PCR product were analyzed for nucleotide sequences of the SARS-CoV spike.

### Expression and purification of RBD of SARS-CoV variants and mutants

Plasmids encoding the RBD (residues 318–510) fused C-terminally with C9 tag were transfected into 293T for expression. All the mutants of RBD were constructed by site-directed mutagenesis. D480A- or D480G-RBD was made using Tor2-RBD as template. Anti-C9 Ab 1D4 (National Cell Culture Center) was conjugated with protein A Sepharose and used for affinity purification of RBD-C9.

### Construction of 80R-Vκ-cs and 80R-fm library

VH (Variable region of heavy chain) gene of 80R was cloned as a NcoI/BspEI fragment into the vector pFarber-Vκ-rep which contains a repertoire of 1.2×10^8^ non-immune Vκ genes derived from 57 healthy donors. Ligated DNA was transformed into eclectroporation-competent *E. Coli*. TG1 cells following manufacture's instructions (Stratagene, La Jolla, CA). Three transformations were performed to generate the 80R-VL-cs-library. For 80R- fm-library, 80R scFv containing pFarber phagemid was used as DNA template. Targeting residues were mutated to all 20 amino acids by using degenerated oligonucleotides contains random NNK codon (N = A+T+G+C and K = G+T) and QuikChange method (Stratagene). The NNK codon encodes all 20 amino acids and UAG stop codon, which can be suppressed in SupE *E.Coli* bacterial strains. Mutated pFarber-80R-scFv DNA was electroporated into TG1 cells to generate 80R-fm-library. Phage antibodies from each libraries were produced as described [40] and used for panning (selection).

### Selection of phage library and screening of S1-RBD-specific phage antibodies

Three human non-immune scFv libraries were used in this study. Two of them (a total of 2.7×10^10^ members) were constructed from peripheral blood B-cells of 57 un-immunized donors in our lab (Mehta I/II libraries), and the third one (2.3×10^10^) was constructed from 47 healthy donors at Fox Chase Cancer Center. 80R-Vκ-cs library and fm-libraries were described as above. 5×10^11^ pfu of phage-scFvs prepared from each library [40] were used for selection of scFvs against different RBD targets separately. Purified RBDs were either coated in maxisorp immunotubes (Nunc, Naperville, IL) or conjugated to magnetic beads (Dynabeads M-270 Epoxy, Dynal Inc.) following manufacturer's instructions. Immunotube-bound or beads-coupled proteins were incubated with phage-scFvs from different libraries. Non-specifically absorbed phages were removed by intensive washings with PBST (PBS containing 0.05% Tween 20). Specific bound phages were eluted with 100 mM triethylamine, neutralized by 1 M Tris pH 7.4, infected into TG1 e.coli., amplified and used for further selections as described previously [40]. Randomly picked single phage-scFv clones were screened for specific binding to different RBD targets by ELISA after two rounds of panning. Clones that bound to targets with *A*_450_>1.0 were selected for further sequence analysis. VH and VL chain of these clones were sequenced and their corresponding amino acid sequences were aligned to identify unique clones.

### Production of phage-scFvs and whole human IgG1s

Phage-scFvs for individual clones were produced for neutralization assay using the same method as making phage library [40]. Phage particles were concentrated 25 times (2–6×10^13^) by using PEG/NaCl precipitation. Whole human IgG1s were produced as described previously [17]. In brief, the VH and VL gene fragments of the selected scFvs were separately sub-cloned into human IgG1 kappa light chain or lambda light chain expression vector TCAE5 or TCAE6 [41]. Human IgG1s were expressed in 293F cells (Invitrogen) or 293T by transient transfection and purified by protein A sepharose affinity chromatography.

### ELISA and competition ELISA

96-well Maxisorp immunoplates were coated with 0.2 µg antigen per well or control proteins. Testing human sera, Abs or phage-Abs in PBS containing 2% nonfat milk were then added. For competition ELISA, competitive sera or Abs were pre-mixed with testing Abs for 30 mins at RT and then added. Specific bound Abs or phage-Abs were detected by adding HRP-conjugated anti-human IgG or HRP-labeled anti-M13, respectively. TMB substrate for HRP was then added, the reaction was stopped 5 mins later, and absorbance at 450 nm was measured.

### Surface Plasmon Resonance (SPR) analysis

Binding of mAbs to various RBDs were anaylyzed on a Biacore T100 (Biacore) at 25°C. Anti-human IgG Fc antibody (Biacore) was covalently coated to CM4 sensor chip by amine-coupling using the coupling kit (Biacore). Abs were captured onto anti-human IgG Fc surfaces at the flow rate of 10 µl/min in HBS buffer (Biacore). RBDs were injected over each flow cell at the flow rate of 30 µl /min in HBS buffer at concentrations ranging from 0.15 to 100 nM for interactions of Abs with Tor2-RBD or GD03-RBD, and 15.6 to 2000 nM for the interaction of Abs with D480A-RBD, respectively. A buffer injection served as a negative control. Upon completion of each association and dissociation cycle, surfaces were regenerated with 3 M MgCl_2_ solution. The association rates (*ka*), dissociation rate constants (*kd*), and affinity constants (*KD*) were calculated using Biacore T100 evaluation software. The goodness of each fit was based on the agreement between experimental data and the calculated fits, where the Chi^2^ values were below 1.0. Surface densities of Abs were optimized to minimize mass transfer. All *ka*, *kd*, *KD* reported here represent the means and standard errors of at least two experiments.

### Statistics

One-way ANOVA for correlated samples was used to measure differences between different types of serum samples in neutralizing pseudo viral infection. Unpaired Student t-test was used for statistic analysis of differences between serum samples in binding to Tor2-RBD and competition ability of 80R's binding to Tor2-RBD.

## Supporting Information

Figure S1 Biosensorgrams of binding analysis of nAbs with spike RBDs.
(0.61 MB DOC)
