# Supplementary material for: Broadening of Neutralization Activity to Directly Block a Dominant Antibody-Driven SARS-Coronavirus Evolution Pathway
Source: PLoS Pathog. 2008 Nov 7;4(11):e1000197. doi: 10.1371/journal.ppat.1000197 (PMC2572002; doi:10.1371/journal.ppat.1000197)

## Supporting Information

**Figure Legends**

**Figure S1. Biosensorgrams of binding analysis of nAbs with spike RBDs.** Abs were captured on a CM4 chip via immobilized anti-human IgG1 on the chip. Serial dilutions of Tor2-, GD03- or D480A-RBD were injected over the chip surface. Binding kinetics was evaluated using a 1:1 interaction model or a two state conformational change model. In each panel, the binding responses (red lines) are overlaid with the fit of the interaction model (black lines). The biosensorgrams were representative examples of two to four independent experiments.

# Fig. S1


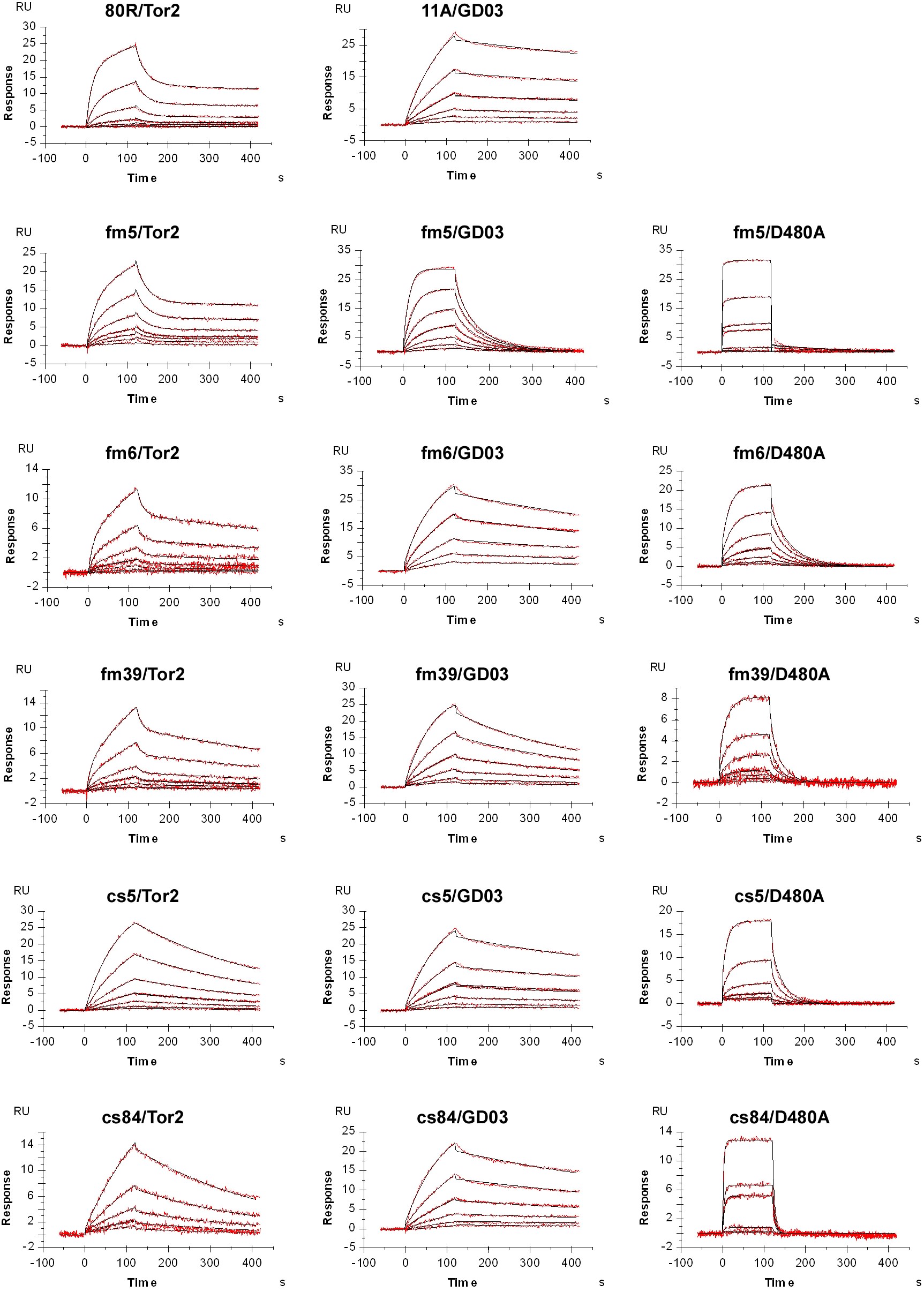

Supplement: Figure S1 — Biosensorgrams of binding analysis of nAbs with spike RBDs. (0.61 MB DOC) [file ppat.1000197.s001.doc]
